# Response of soil N_2_O emission and nitrogen utilization to organic matter in the wheat and maize rotation system

**DOI:** 10.1038/s41598-021-83832-7

**Published:** 2021-02-23

**Authors:** Xiaoxiao Shu, Yanqun Wang, Yaling Wang, Yang Ma, Mingxin Men, Yunpu Zheng, Cheng Xue, Zhengping Peng, Christos Noulas

**Affiliations:** 1 Institute of Land Engineering and Technology, Shaanxi Provincial Land Engineering Construction Group Co., Ltd., Xi’an, 710075 China; 2 College of Resources and Environmental Sciences/Hebei Province Key Laboratory for Farmland Eco-Environment, Hebei Agricultural University, Baoding, 071000 China; 3 State Key Laboratory of North China Crop Improvement and Regulation, Baoding, 071000 China; 4 School of Water Conservancy and Hydropower, Hebei University of Engineering, Handan, 056000 China; 5 Department of Soil and Water Resources, Institute of Industrial and Forage Crops, Hellenic Agricultural Organization—“Demeter”, 41335 Larissa, Greece

**Keywords:** Climate sciences, Ecology, Environmental sciences, Environmental social sciences

## Abstract

The appropriate nitrogen (N) fertilizer regulator could increase N utilization of crops and reduce N losses in the North China Plain. We investigated the effects of reduced inorganic-N rate combined with an organic fertilizer on nitrous oxide (N_2_O) emissions in winter wheat and summer maize rotation system. Simultaneously studied the effect of different treatments on N use efficiency (NUE), N balance and net income. After reducing the amount of nitrogen fertilizer in the wheat-corn rotation system, the results showed that the cumulative emission of soil N_2_O from the RN40% + HOM [40% of RN (recommended inorganic-N rate) with homemade organic matter] treatment was 41.0% lower than that of the RN treatment. In addition, the N production efficiency, agronomic efficiency, and apparent utilization were significantly increased by 50.2%, 72.4% and 19.5% than RN, respectively. The use of RN40% + HOM resulted in 22.0 and 30.1% lower soil N residual and N losses as compared with RN. After adding organic substances, soil N_2_O cumulative emission of RN40% + HOM treatment decreased by 20.9% than that of the HAN (zinc and humic acid urea at the same inorganic-N rate of RN) treatment. The N production efficiency, N agronomic efficiency and NUE of RN40% + HOM treatment were 36.6%, 40.9% and 15.3% higher than HAN’s. Moreover, soil residual and apparent loss N were 23.3% and 18.0% less than HAN’s. The RN40% + HOM treatment appears to be the most effective as a fertilizer control method where it reduced N fertilizer input and its loss to the environment and provided the highest grain yield.

## Introduction

An aspect of climate change is global warming and ozone depletion caused by greenhouse gas (GHG) emissions. In China, relatively recent data demonstrated that average surface temperature has been ascended 0.91 °C in the past 100 years, which is higher than the global increase of 0.85 °C^1^. It showed that the average warming over China would increase by approximately 1.8 and 2.6 °C under the global 1.5 and 2.0 °C target by analyzing 22 models consistently. These results suggest that the warming over China is faster than the global mean. Furthermore, the warming shows a clear spatial distinction over China being stronger in the northwest part and weaker in the southeast part^2^. Nitrous oxide (N_2_O) is a type of GHG, which has an temperature increasing potential of 298 times compared to CO_2_ and 25 times to CH_4_^3^. The annual release of N_2_O–N from soil is 1.7–4.8 Tg, more than 61% of the total global emissions^4^, and the source is mainly resulted from synthetic nitrogen (N) fertilizers^5^. Although the use of synthetic N fertilizers have promoted crop production, undersirable consequences including increased N_2_O emissions have been reported^6^. Therefore, rational application of N fertilizers and reduction of N_2_O emissions from farmlands are positive steps towards mitigating future climate change.

North China Plain is a main crop production area with winter wheat/summer maize rotation. Nitrogen is the primary nutrient element that limiting plant growth. It is also essential for economic and environmental effectiveness besides its agronomic value^7,8^. Farmers in some high-yielding areas apply extremely high N potions reaching up to 600 kg N ha^−1^^9^. Consequently, the N use efficiency (NUE) was only between 30 and 35% with a high loss rate of 45%^10^. Low NUE may be due to N leaching through the soil–plant system^11^ and the reduction in nitrogen uptake and utilization as a major components of NUE^12^. Fertilizer N application generally affected N_2_O emission with a loss ratio from 0.48 to 0.96%^13,14^. In addition, excessive N fertilizer entered into the environment through nitrate leaching and other gaseous losses^15,16^. Therefore, investigating N fate in the soil–plant–atmosphere system is critical for assessing the optimal N fertilizer application to coordinate both crop growth and environmental protection^8^.

In order to mitigate N_2_O emission from agricultural ecosystems, several technical measures have already been proposed, such as the use of fertilizers containing nitrification inhibitors^17^, organic fertilizer instead of chemical fertilizer^18^ and protectiveness tillage^19^. Overuse of chemical N fertilizer fails to bring crop yield potential into full play, and also easily lost N in gaseous or other forms^19^. Nutrients release in organic fertilizer is slow which reduces N leaching and is more conducive to crop growth than chemical N fertilizer^18^. The combination of microbial agents with N fertilizer has a positive effect on soil structure, fertility and yield, and therefore jointly applications of microbial fertilizer and chemical fertilizer can also reduce N_2_O emission^20,21^.

However, very few studies have focused on the jointly application of organic–inorganic compound fertilizer, biological bacterial fertilizer, and inorganic fertilizer in the grain field. Therefore, this study investigated the effects of reduced inorganic-N rate combined with homemade organic matters on N_2_O emissions, NUE, N balance and net income in winter wheat and summer maize rotation system. The main objective was to determine the appropriate N fertilizer regulator to reduce N environmental risks and enhance N effects during wheat and maize productions in NCP.

## Materials and methods

### Study site

**Figure 1:**
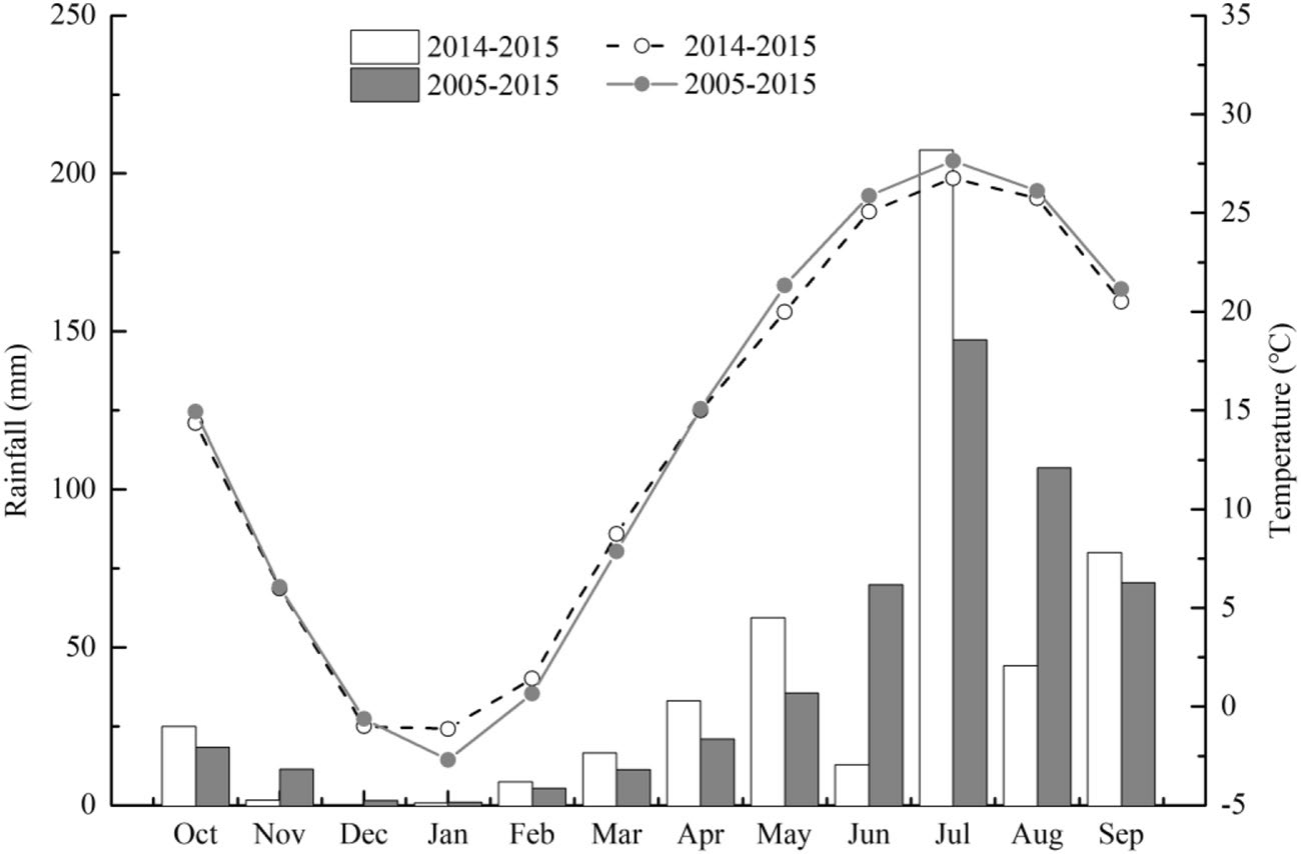
Monthly precipitation and average temperature during the experimental year (2014–2015) and the mean values in the last ten years (2005–2015) in the test area. Data was from meteorological station.

The study site (N 38° 49′, E 115° 26′) is located in the Guanzhuang Village in Baoding City of Hebei Province, China, in the humid temperate and monsoon climatic zone with the average annual air temperature of 13 °C, annual rainfall of 500 mm, and frost-free period is 210 days. Although the experiment was a one-year, the distribution of precipitation (488.50 mm) and temperature (13.45 °C) during the experimental period (2014–2015) were close to the the latest 10-year averages (2005–2015) (500.19 mm and 13.61 °C) (Fig. 1). Determine the basic nutrient indexes of the 0–20 cm surface soil in the test plot. The soil type is silty loam, consists with 22.55% sand, 71.09% silt and 6.36% clay. Analysis of soil basic characteristics showed that it has a pH of 8.3, and its content of organic matter, total N, available phosphorus (P) and available potassium (K) was 11.27 g kg^−1^, 1.47 g kg^−1^, 25.49 mg kg^−1^ and 127.43 mg kg^−1^, respectively.

### Experiment materials

The planting mode of the experimental site was a winter wheat-summer maize rotation, the winter wheat variety was ‘Jinnong 6’ that verage thousand weight was 47.6 g. The summer maize variety was ‘Zhengdan 958′ that average thousand weight 330 g.

Test fertilizers include inorganic fertilizer, organic fertilizer, soil conditioner, compound bateria, amino acid liquid fertilizer and nutrient agent. Inorganic N, P and K in the tested fertilizers were provided by urea (N 46%), superphosphate (P_2_O_5_,16%) and potassium chloride (K_2_O, 54%), respectively, as well as in the form of zinc and humic acid urea which is mainly a combination of N with humic acid (N 46% and HA 1.2%). The organic fertilizer used in the experiment was mainly decomposed chicken manure. But the N content in the chicken manure is 1.32% in wheat season and 4.48% in maize sason. Soil conditioner mainly containing calcium (Ca) and magnesium (Mn), Compound bacteria could fix N potentially, promote root growth, decompose cellulose lignin and thus rapidly to degrade. The number of living bacteria reached 2 billion per gram. Amino acid liquid fertilizer and nutrient agent sprayed according to crop growth to provide the required amino acids and trace elements for plant growth.

### Experiment design

Field experiment consisted of five treatments with 3 replicates. The experiment uses a completely randomized block setting, the plot size was 79.2 m^2^ (13.2 m × 6 m). Before the experiment, no crops were planted in the area and it was idle for more than one year. The five treatments were: CK (zero N), FN (farmers’ traditional inorganic N rate, through mass surveys on actual production), RN (recommended inorganic-N rate, according to the experimental results of many scholars, combined with the local soil N supply, crop straw returning in the previous season as well as wheat or maize N demand for target yield in the current season)^22,23^, HAN (zinc and humic acid urea, the N supply same as RN), RN40% + HOM (40% inorganic N rate of RN (RN40%) with homemade organic matters (HOM). HOM was an organic control measure, it including organic fertilizer, soil conditioner compound bacteria, amino acid liquid fertilizer and nutrient agent. these constituents and amount according to Shu et al^24^ (Table 1).

**Table 1:** Rates of pure N and organic matters in different fertilization treatments.

Treatment^a^	Total nutrient(kg ha^−1^)				Other matters of wheat and maize (kg ha^−1^ or L ha^−1^ for liquid fertilizers)				
	Wheat		Maize		Organic fertilizer	Soil conditioner	Compound bacteria	Amino acid liquid fertilizer	Nutrient agent
	N	HA	N	HA					
CK	0	0	0	0	0	0	0	0	0
FN	285	0	390	0	0	0	0	0	0
RN	225	0	300	0	0	0	0	0	0
HAN	225	0.27	300	0.36	0	0	0	0	0
RN40% + HOM	145	0	254	0	3000	225	30	4.5	4.5

^a^Treatment: CK (zero N), FN (farmers’ traditional inorganic N rate), RN (recommended inorganic-N rate), HAN (zinc and humic acid urea), RN40% + HOM (40% inorganic N rate of RN with homemade organic matters).

For wheat, N fertilizer was broadcast for ratio of 4:3 (basal to topdressing) in RN40% + HOM, whereas for the rest N treatments the ratio was 1:1. During maize planting, N ratio (basal to topdressing) for all treatments was 2:3.The N, P and K fertilizers for wheat were applied in the form of urea, single superphosphate and potassium chloride, respectively. The amount of N fertilizer applied in different treatments of different crops is different, specific application amount reference Table 1. Except for treatment RN40% + HOM, all treatments have the same amount of single superphosphate and potassium chloride. Single superphosphate (120 kg P_2_O_5_ ha^−1^)and potassium chloride (150 kg K_2_O ha^−1^)were used in winter wheat season. For maize, single superphosphate (90 kg P_2_O_5_ ha^−1^) and potassium chloride (150 kg K_2_O ha^−1^) were used. P and K fertilizers were applied once before sowing. For the doses of P and K in RN40% + HOM brought by organic fertilizer were firstly assessed (48.4 kg P_2_O_5_ ha^−1^ and 149.3 kg K_2_O ha^−1^ for winter wheat; 87.2 P_2_O_5_ ha^−1^ and 19.1 kg K_2_O ha^−1^ for maize), remaining amounts were supplemented with chemical P and K fertilizers.

Wheat at a rate of 187.5 kg ha^−1^ with a row space of 15 cm, was sown on 12 October 2014 and harvested at 7 June 2015. Then, at the same wheat plot, Maize of 37.5 kg ha^−1^ with a row space of 57 cm, was sown on 18 June 2015 and harvested at 5 October 2015.

### N_2_O sampling and measurements

N_2_O gas was collected using a closed static chamber from sowing to harvest of wheat and maize^25^. The sampling box was divided into two parts and made by PVC material: a box body and a base. The upper part of the box body was provided with a gas sampling port sealed with a rubber plug, and a thermometer probe was arranged inside the box body to monitor the soil surface temperature. The box body is 15 cm high and the bottom diameter is 25 cm. The base was ring shaped, and buried into the soil. Gas collection was performed from 9:00 to10:00 am. A 30 mL of air sample was collected at 0, 8, 16 min after closure^26^. The air samples were taken once at an interval of 7 days in general and subsequently continuous 5 days following fertilization or precipitation. Continue to collect gas samples from the beginning of the experiment. No gas samples are collected during the freezing of wheat field soil in February and March every year. At the same time, the air temperature was measured by a thermometer and the soil moisture in the 0–5 cm depth was measured by soil moisture tester (TK3-BASIC). Gas concentrations were analyzed by using a gas chromatography (Agilent 7890 A, USA), fitted with a 4 mm by 3 m stainless steel column packed with Porapack Q and N_2_ was used as the carrier gas. The column and the detector temperatures were set at 70 °C and 300 °C, respectively. The standard N_2_O was supplied from National Center of Standard Measurement.

N_2_O flux was calculated using the following equation (Wang et al.^27^).

1 $${\text{F}} = \rho \times {\text{H}} \times T_{0} \frac{{\left( {c_{2} /T_{2} - c_{1} /T_{1} } \right)}}{\Delta t}$$

where F is N_2_O emission flux, ρ = m/22.414, ρ is the density of gas in airtight box, m is molecular weight, H is the height of the static chamber, *T*_*0*_ is 273 K, c_1_ and c_2_ are the gas concentration in time of *t*_1_and *t*_2_, respectively, *T*_1_and *T*_2_ are gas temperatures, ∆t = *t*_2_ − *t*_1_ ,where *t*_2_ and *t*_1_are times.

Cumlative N_2_O emissions were from the growth season was calculated by the equation:

2 $${\text{T = }}\sum {\left[ {{{\left( {{\text{F}}_{{\text{i + 1}}} {\text{ + F}}_{{\text{i}}} } \right)} \mathord{\left/ {\vphantom {{\left( {{\text{F}}_{{\text{i + 1}}} {\text{ + F}}_{{\text{i}}} } \right)} {2}}} \right. \kern-\nulldelimiterspace} {2}}} \right]} \times \left( {{\text{D}}_{{\text{i + 1}}} - {\text{D}}_{{\text{i}}} } \right) \times {{{24}} \mathord{\left/ {\vphantom {{{24}} {{1000} \times {667} \times {15} \times {10}^{{ - {6}}} }}} \right. \kern-\nulldelimiterspace} {{1000} \times {667} \times {15} \times {10}^{{ - {6}}} }}$$

where T is the total amount of N_2_O emissions from the growth stage (kg N ha^−1^), F_i_ and F_i+1_ denote the N_2_O flux of the i and i + 1 sub-sampling (μg N m^−2^ h^−1^); D_i_ and D_i+1_ represent sampling days (d)^26^.

N_2_O emission coefficient (EF) was estimated with equation^28^:

3 $$EF(\% ) = \left[ {\left( {{\text{Cumulative}}\;{\text{N}}_{{2}} {\text{O}}\;{\text{emissions}}\;{\text{from}}\;{\text{fertilized}}\;{\text{plots}} - {\text{control}}\;{\text{plots}}} \right)/{\text{N}}\;{\text{fertilizer}}\;{\text{rate}}} \right] \times 100$$

### Soil sampling and measurements

At wheat and maize maturity, soil samples were collected from depths of 0–20, 20–40 and 40–60 cm with a hand probe from three places in central rows of each plot and mixed together. Fresh soil samples were sieved through a 2 mm, extracted with 1 mol L^−1^ KCl and a soil-solution ratio of 1:10, and analyzed for inorganic N (mainly including NH_4_^+^–N and NO_3_^−^–N) contents with continuous flow analysis technique(AA3-HR, Germany)^22^. Soil moisture and density of each soil layer were measured simultaneously, and the soil residual N in 0–60 cm was calculated. Other soil sample was air-dried and sieved, organic matter and total N content were measured by agrochemical analysis method^29^.

### Plant harvest

For wheat, plants with double rows (1 m length) in each plot were harvested and 20 spikes were selected to count the numbers of effective spikes. All the harvested plant samples were separated into straw (including stem, leaves and remaining of ears) and grains, and the grain yield was calculated to 12.5% moisture content (PM-8188, Japan). Three samples were chosen from each plot and weighted to get the average 1000-grain weight.

For maize, two representative plants in each plot were harvested and separated into straw (including stems, leaves, tassels, husks, cobs) and grains in the central rows. Moreover, 20 ears were continuously selected to thresh and measured grain yield. Grain yield was calculated to 14% moisture content (PM-8188, Japan).

All harvested wheat and maize samples were dried, weighed, ground into powder to measure the total N content using H_2_SO_4_–H_2_O_2_ Kjeldahl digestion method^29^.

### N balance and N efficiencies

Total N input was comprised of N fertilizer, the initial inorganic N in soil before planting (including both NO_3_^−^–N and NH_4_^+^–N), pre-crop N straw return (no straw was returned when sowing wheat and the N uptake in maize stage was calculated from pre-wheat straw), N deposition from dry and wet atmosphere and mineralized N in soil. Atmospheric N deposition was derived from Research result by Liu et al.^30^. N output was comprised of crop uptake, post-harvest residual soil N and apparent N loss. This study calculated soil N to a depth of 0–60 cm. Mineralized soil N, apparent N loss, Nitrogen production effiency (NPE) , Nitrogen agronomic effiency (NAE) and Nitrogen use efficiency (NUE) were calculated as follows:

4 $$\begin{aligned} {\text{Mineralized}}\;{\text{N}}\left( {{\text{kg}}\,{\text{ha}}^{ - 1} } \right) & = {\text{Crop}}\;{\text{N}}\;{\text{uptake}}\;{\text{in}}\;{\text{CK}} + {\text{Post - harvest}}\;{\text{residual}}\;{\text{soil}}\;{\text{N}}\;{\text{in}}\;{\text{CK}}{-}{\text{Pre - planting}}\;{\text{soil}}\;{\text{N}}\;{\text{in}}\;{\text{CK}} \\ & \quad {-}{\text{N}}\;{\text{deposition}}\;{\text{from}}\;{\text{atmosphere}}\;{\text{in}}\;{\text{CK}} - {\text{Pre - crop}}\;{\text{straw}}\;{\text{return}}\;{\text{N}}\;{\text{in}}\;{\text{CK}} \\ \end{aligned}$$

5 $${\text{Apparent}}\;{\text{N}}\;{\text{loss}}\left( {{\text{kg}}\,{\text{ha}}^{ - 1} } \right) = {\text{Total}}\;{\text{N}}\;{\text{input}} - {\text{crop}}\;{\text{N}}\;{\text{Uptake}} - {\text{post - harvest}}\;{\text{residual}}\;{\text{soil}}\;{\text{N}}$$

6 $${\text{NPE}}\left( {{\text{kg}}\,{\text{kg}}^{ - 1} } \right) = {\text{Plant}}\;{\text{yield}}/{\text{N}}\;{\text{fertlizer}}\;{\text{rate}}$$

7 $${\text{NAE}}\left( {{\text{kg}}\,{\text{kg}}^{ - 1} } \right) = \left[ {\left( {{\text{Plant}}\;{\text{yield}}\;{\text{with}}\;{\text{N}}\;{\text{application}} - {\text{plant}}\;{\text{yield}}\;{\text{without}}\;{\text{N}}\;{\text{fertlizer}}} \right)/{\text{N}}\;{\text{fertlizer}}\;{\text{rate}}} \right]$$

8 $${\text{NUE}}\left( \% \right) = \left[ {\left( {{\text{N}}\;{\text{content}}\;{\text{in}}\;{\text{plant}}\;{\text{with}}\;{\text{N}}\;{\text{fertilizer}}{-}{\text{N}}\;{\text{content}}\;{\text{in}}\;{\text{plant}}\;{\text{without}}\;{\text{N}}\;{\text{fertilizer}}} \right)/{\text{N}}\;{\text{fertlizer}}\;{\text{rate}}} \right] \times 100$$

### Net income analyses

Prices of fertilizers and grains as well as other costs in Chinese Yuan (RMB: 1 USD = 6.71 RMB in the experiment year) were based on local prices. Net income was calculated by the equation:

9 $${\text{Net}}\;{\text{income}} = {\text{Output}}\;{\text{value}}{-}{\text{fertilizer}}\;{\text{cost}}{-}{\text{other}}\;{\text{field}}\;{\text{management}}\;{\text{costs}}$$

10 $${\text{Output}}\;{\text{value}} = {\text{Grain}}\;{\text{yield}} \times {\text{grain}}\;{\text{price}}$$

where Fertilizer costs were composed of the prices of inorganic N (3.9 RMB kg^−1^), P_2_O_5_ (5.65 RMB kg^−1^), K_2_O (6.5 RMB kg^−1^), pure N in zinc and humic acid urea (5.0 RMB kg^−1^), decomposed chicken manure (0.5 RMB kg^−1^), soil conditioner (2.8 RMB kg^−1^), compound bacteria, amino acid liquid fertilizer and nutrient agent together (30 RMB kg^−1^). Other field management costs included seed, labor for fertilization, irrigation, mechanical sowing, etc. Grain prices of wheat and maize during the experiment were 2.2 and 1.8 RMB kg^−1^, respectively.

### Statistical analysis

This research adopted SPSS Statistics 20.0 software (SPSS Inc., Chicago, IL, USA) to date analysis. Through least significant differences (LSD) method, the statistically significant differences were calculated. The differences level was prominent when *P* < 0.05. Spearman method was used to analyze the correlation between measured variables and N_2_O flux^31^. Besides, the difference level is considered extremely significant when *P* < 0.01. All the figures and statistical analyses were computed in Origin 9.0 (Origin Lab Ltd., Guangzhou, China).

## Results

### N_2_O emission characteristics in wheat season

**Figure 2:**
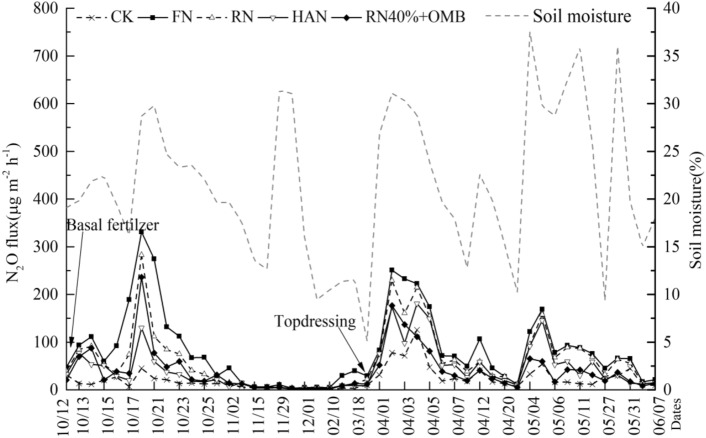
N_2_O emission characteristics of the wheat. Treatment: CK (zero N), FN (farmers’ traditional inorganic N rate), RN (recommended inorganic-N rate), HAN (zinc and humic acid urea), RN40% + HOM (40% inorganic N rate of RN with homemade organic matters).

Average N_2_O emission flux in N treatments was 2.0–4.3 times of CK (Fig. 2). Enhancing N fertilizer marginally increased soil N_2_O flux. In all N treatments the N_2_O emission flux fluctuated from 0.5 to 330.8 μg m^−2^ h^−1^ for wheat. The peaks of N_2_O emission mainly occurred after basal fertilization, irrigation, and rainfall events. Within 7 days after wheat sowing (10/12), the average daily soil N_2_O emission flux achieved the first peak of 330.8 μg m^−2^ h^−1^(FN). The basal fertilization resulted in an increase of soil N_2_O emission (Fig. 2). Compared to FN, soil N_2_O emission peaks of RN, HAN and RN40% + HOM were decreased by 14.6%, 60.6% and 28.6%, respectively. The N_2_O emission gradually increased due to N topdressing, rainfall and temperature rising during the vegetative stage of wheat (March 2015). The second N_2_O emission peak appeared after N fertilizer topdressing and FN exhibited the highest soil N2O emission (> 220 μg m^−2^ h^−1^ during three consecutive dates) (Fig. 2). With the gradual increase in temperature (after May 2015), the growth rate of wheat has accelerated, the growth of wheat has absorbed more nitrogen, and the N_2_O emission flux has slowly decreased. However, after the rainfall, the moisture increased sharply, and the third N_2_O emission peak occurred (Fig. 2).

### N_2_O emission characteristics in maize season

**Figure 3:**
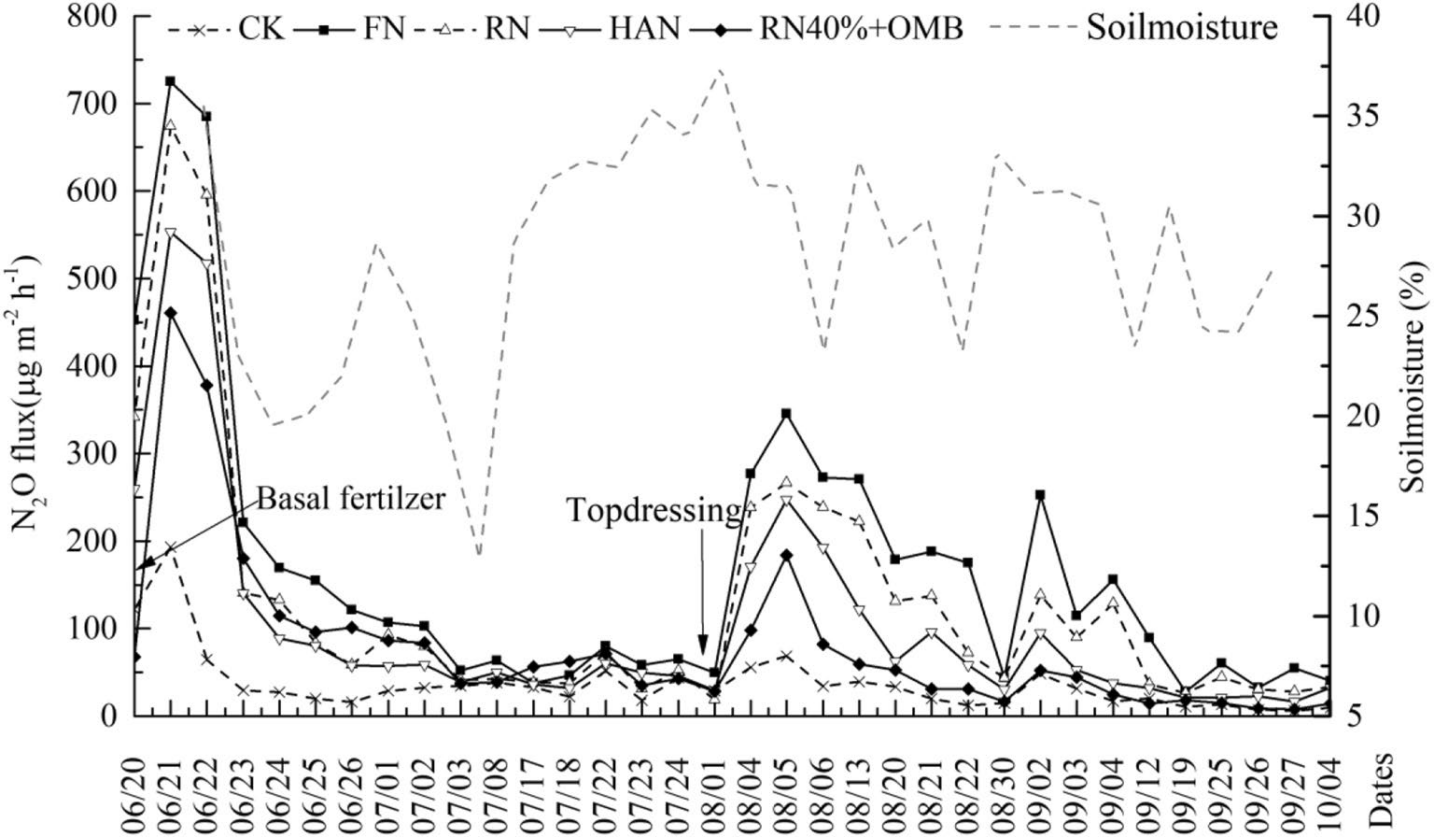
N_2_O emission characteristics of the maize. Treatment: CK (zero N), FN (farmers’ traditional inorganic N rate), RN (recommended inorganic-N rate), HAN (zinc and humic acid urea), RN40% + HOM (40% inorganic N rate of RN with homemade organic matters).

For maize, the peak occurrence of N_2_O emission was basically consistent with wheat. Soil N_2_O emission peaked on the 4th day after sowing and ranged from 193.2 μg m^−2^ h^−1^ (CK) to 725.1 μg m^−2^ h^−1^ (FN). Soil N_2_O emission rates from RN, HAN and RN40% + HOM were 7.0%, 23.7% and 36.5% lower than FN, respectively (Fig. 3). The second N_2_O emission peak appeared after N topdressing (Fig. 3). The peak value of each treatment decreased in the range of 52.3–60.5% compared to the first peak value of the respective N treatments. N_2_O emission peak rates in HAN and RN40% + HOM were decreased by 7.1% and 30.9% compared to RN, respectively. Subsequent N_2_O emission rates exhibited lower peaks and were usually recorded after heavy rain events, at maturity the average N_2_O emission flux dropped below 90 μg m^−2^ h^−1^. For maize, the average N_2_O emission fluxes in HAN and RN40% + HOM were reduced by 24.2% and 38.0% compared to RN during the whole growth period, respectively.

### Cumulative N_2_O emissions from soil and correlation analysis of environmental factors

In the wheat and corn seasons, the cumulative N_2_O emission change trend of each treatment is consistent. And the emissions from the corn season are greater than those from the wheat season (Table 2). The cumulative emissions are FN > RN > HAN > RN40% + HOM > CK,were significantly related to the amount of inorganic nitrogen fertilizer. The cumulative N_2_O emissions during the whole study period were ranged from 1.30 kg ha^−1^ (CK) to 5.29 kg ha^−1^ (FN) (Table 2). The cumulative N_2_O of FN treatment was the highest among all treatments due to the high N amount. Soil cumulative N_2_O emissions in RN40% + HOM was significantly lower by 41.0% in the rotation system as compared to RN. Compared to HAN, Soil cumulative N_2_O emission of in RN40% + HOM was significantly decreased by 20.9% (Table 2).

**Table 2:** N_2_O cumulative emission and its influencing factors.

Crop	Treatment^a^	Cumulative emission (kg ha^−1^)	EF (%)	Correlation with temperature (°C)	Correlation with moisture (%)
Winter wheat	CK	0.55d	–	0.153	0.436**
	FN	2.08a	0.34a	0.094	0.534**
	RN	1.47b	0.26ab	0.125	0.576**
	HAN	1.11c	0.16b	0.103	0.541**
	RN40% + HOM	0.95c	0.18b	0.035	0.496**
Summer maize	CK	0.75e	–	0.332	0.561**
	FN	3.21a	0.40a	0.206	0.515**
	RN	2.38b	0.35b	0.186	0.524**
	HAN	1.76c	0.21c	0.2	0.499**
	RN40% + HOM	1.32d	0.14d	0.269	0.373*
Rotation	CK	1.30e	–	0.292**	0.535**
	FN	5.29a	0.38a	0.285**	0.537**
	RN	3.85b	0.31b	0.272*	0.537**
	HAN	2.87c	0.19c	0.273*	0.506**
	RN40% + HOM	2.27d	0.15c	0.242*	0.449**

^a^Treatment: CK (zero N), FN (farmers’ traditional inorganic N rate),RN (recommended inorganic-N rate), HAN (zinc and humic acid urea), RN40% + HOM (40% inorganic N rate of RN with homemade organic matters). Different letters in the same column within the same crop and rotation system represent significant differences of mean values at the *P* < 0.05 level. * and ** indicate significant correlations at 0.05 and 0.01 level, respectively (n = 82).

Regardless of the wheat season or the corn season, the change trend of EF is consistent with the change of cumulative N_2_O emissions (Table 2). Moreover, the N_2_O emission intensity gradually increased with fertilizer rate. EF of reducing N treatments compared to FN was decreased by 0.07–0.23% in the wheat/maize rotation system. The treatments of RN40% + HOM were significantly decreased by 0.16% in the wheat/maize rotation system as compared to RN. But it was close to HAN among whole growth stage.

It was showed that soil N_2_O emission was significantly correlated with soil moisture in two seasons and wheat/maize rotation system (Table 2). The correlation between soil temperature and soil N_2_O flux was also significant in the whole wheat/maize rotation system but weaker in single wheat or maize season.

### Effects of organic matter addition on N uptake and efficiency

N fertilizer increased grain N uptake compared with CK in wheat, maize and wheat/maize rotation system (Table 3). Grain N uptake was higher from 20.5 kg ha^−1^ (FN) to 65.9 kg ha^−1^ (RN40% + HOM) in wheat, from 64.1 kg ha^−1^ (FN) to 94.7 kg ha^−1^ (HAN) in maize and from 84.6 kg ha^−1^ (FN) to 155.7 kg ha^−1^ (RN40% + HOM) in the wheat/maize rotation system as compared to CK. Compared with FN treatment, although other N treatments reduced the amount of nitrogen fertilizer, the nitrogen uptake of its grains increased by 28.4% (RN), 29.4% (HAN), and 35.7%(RN40% + HOM) in the wheat/maize rotation respectively. Similar trend were observed for plant N uptakes. Plant N uptakes were also enhanced from 45.5 kg ha^−1^ (FN) to 87.5 kg ha^−1^ (RN40% + HOM), 111.5 kg ha^−1^ (FN) to 147.6 kg ha^−1^ (HAN) and 157.0 kg ha^−1^ (FN) to 221.4 kg ha^−1^ (RN40% + HOM) in wheat, maize and wheat/maize rotation system, respectively. Compared with FN, other N treatments raised N uptake in plant by 8.7% (RN), 14.7% (HAN) and 17.5% (RN40% + HOM) in the wheat/maize rotation system respectively.

**Table 3:** N uptake and efficiency under the regulation of organic regulation.

Crop	Treatment^a^	Grain N (kg ha^−1^)	Plant N (kg ha^−1^)	NPE (kg kg^−1^)	NAE (kg kg^−1^)	NUE (%)
Wheat	CK	41.9b	80.4c	–	–	–
	FN	62.4b	125.9b	25.0d	10.3d	13.6c
	RN	89.2a	142.2ab	32.5c	13.8c	27.5b
	HAN	90.4a	143.9ab	35.2b	16.5b	28.2b
	RN40% + HOM	107.8a	167.9a	55.4a	26.4a	60.3a
Maize	CK	72.9b	131.2b	–	–	–
	FN	137.0a	242.7a	19.9d	9.6d	28.6b
	RN	166.9a	258.5a	26.6c	13.2c	42.4ab
	HAN	167.6a	278.8a	29.7b	16.3b	49.2a
	RN40% + HOM	162.7a	265.1a	37.1a	21.2a	52.7a
Rotation system	CK	114.8c	211.6b	–	–	–
	FN	199.4b	368.6b	22.1d	9.9d	23.3c
	RN	256.1a	400.7ab	29.1c	13.4c	36.0bc
	HAN	258.0a	422.7ab	32.0b	16.4b	40.2b
	RN40% + HOM	270.5a	433.0a	43.7a	23.1a	55.5a

^a^Treatment: CK (zero N), FN (farmers’ traditional inorganic N rate),RN (recommended inorganic-N rate), HAN (zinc and humic acid urea), RN40% + HOM (40% inorganic N rate of RN with homemade organic matters). Different letters in the same column within the same crop and rotation system represent significant differences of mean values at the *P* < 0.05 level. NPE is N production efficiency. NAE is N agronomic efficiency. NUE is N use efficiency.

In the wheat/maize rotation system, the average NPE, NAE, and NUE of other N treatments were increased by 31.7–97.7%, 35.4–133.3% and 57.1–138.2% respectively compared with FN (Table 3). The NPE in RN40% + HOM increased by 70.5%, 39.5% and 50.2% in wheat, maize and whole wheat/maize rotation system respectively in relation to RN. Correspondingly, the NPE increased by 57.4% and 24.9% in wheat and maize seasons, respectively, and by 36.6% in wheat/maize rotation system than HAN treatment. In the wheat season, corn season, and the whole crop rotation season, the NAE changes in each treatment were consistent with NPE. The NAE of RN + HOM treatment increased by 72.4% and 40.9% compared with RN treatment and HAN treatment in wheat/maize rotation system, respectively. Furthermore, the NUE of the whole wheat/maize system in RN40% + HOM were significantly increased by 19.5% and 15.3%, respectively than RN and HAN.

### N balance under the cropping systems

N fertilizer input of each treatment accounted for 52.3% (RN40% + HOM) to 68.3% (FN) of the total N input in the first season of wheat, accounted for 55.4% (RN40% + HOM) to 60.2% (FN) in maize (Table 4). Compared with FN, the ratio of wheat N uptake to N input in RN, HAN and RN40% + HOM was increased by 9.6–30.3%, soil residual N was decreased by 21.8–43.3%, and the apparent N loss was reduced by 28.3–74.9%. The proportion of N absorbed by crops in RN, HAN and RN40% + HOM was increased by 12.1–20.3% than FN. Meanwhile, the residual soil N was decreased by 46.6%-59.0%, and the apparent N loss was decreased by 18.5–43.0% in maize season.

**Table 4:** N balance in the soil depth of 0–60 cm under the organic matter regulation.

Crop	Treatment^a^	N input (kg ha^−1^)					N output (kg ha^−1^)		
		N fertilizer	Soil N pre-planting	Pre-wheat straw N	N deposition	Mineralized N	Crop uptake	Soil residual	Apparent N loss
Wheat	CK	0	113.2	0	12.8	6.5	80.4	52.1	–
	FN	285	113.2	0	12.8	6.5	125.9	115.2	176.4
	RN	225	113.2	0	12.8	6.5	142.2	88.9	126.4
	HAN	225	113.2	0	12.8	6.5	143.9	90.1	123.5
	RN40% + HOM	145	113.2	0	12.8	6.5	167.9	65.3	44.3
Maize	CK	0	52.1	38.6	15.0	64.2	131.2	38.7	–
	FN	390	115.2	63.5	15.0	64.2	242.7	233.5	171.7
	RN	300	88.9	53.0	15.0	64.2	258.5	122.7	139.9
	HAN	300	90.1	53.5	15.0	64.2	278.8	124.8	119.2
	RN40% + HOM	254	65.3	60.1	15.0	64.2	265.1	95.7	97.8

^a^Treatment: CK (zero N), FN (farmers’ traditional inorganic N rate), RN (recommended inorganic-N rate), HAN (zinc and humic acid urea), RN40% + HOM (40% inorganic N rate of RN with homemade organic matters). The straw returning to the field used the amount of N absorbed by the straw of the previous season.

Total N input of two season crops in FN was 1065.4 kg ha^−1^, which was 1.21–1.45 times higher than the rest treatments with reduced N fertilizer rate. The respective apparent N loss in FN was 1.23–3.98 times higher than the other N treatments. The N input of HAN was similar to RN in wheat or maize, and the apparent N loss in maize crop was reduced by 14.8% after changing the fertilizer type (HAN instead of RN). Compared with RN, the residual soil N of RN40% + HOM was reduced by 22.0% after maize harvested. For the same N treatments in maize the N loss decreased by 30.1%, and the N fertilizer input of two seasons was decreased by 126 kg ha^−1^ in RN40% + HOM as compared to RN. Accordingly, compared with HAN, the residual soil N and apparent N loss in RN40% + HOM were decreased by 23.3% and 18.0%, respectively.

### Analysis of crop economic benefits under organic matter regulation

N fertilization raised the input costs (fertilizer, management), and increased yield and net income (Table 5). Lower fertilizer and management costs were recorded in wheat as compared to maize. CK has the lowest input costs (fertilizer, management), the fertilizer cost and management cost of RM + HOM treatment are higher than others due to the addition of organic matter. Regardless of the wheat season, maize season or the whole crop rotation season, the RM + HOM treatment has the highest yield, and the yield of each treatment is RN40% + HOM > HAN > RN > FN > CK. The yield of RM + HOM treatment significantly increased by 11.1%, 18.0% and 14.7% in wheat season, maize season and wheat/maize rotation season compared with RN treatment.

**Table 5:** Net income of crop rotation system under the organic matter regulation.

Crop	Treatment^a^	Fertilizer cost (USD ha^−1^)	Management cost (USD ha^−1^)	Yield (kg ha^−1^)	Output value (USD ha^−1^)	Net income (USD ha^−1^)
Wheat	CK	246.3	592.4	4206.5c	1379.2e	540.4e
	FN	412.0	655.0	7129.1b	2337.4d	1270.4c
	RN	377.1	655.0	7232.8b	2371.4c	1339.3b
	HAN	414.0	655.0	7912.8a	2594.4b	1525.4a
	RN40% + HOM	799.2	679.6	8034.9a	2634.4a	1155.6d
Maize	CK	221.1	760.1	4024.6c	1079.6e	98.5e
	FN	447.8	822.7	7769.2b	2084.1d	813.7d
	RN	395.5	822.7	7981.0b	2141.0c	922.8b
	HAN	444.6	822.7	8913.0a	2391.0b	1123.7a
	RN40% + HOM	782.6	839.2	9419.2a	2526.8a	904.9c
Rotation system	CK	467.4	1352.5	8231.1c	2458.8e	638.9e
	FN	859.8	1477.6	14,898.3d	4421.5d	2084.1c
	RN	772.6	1477.6	15,213.8b	4512.4c	2262.1b
	HAN	858.6	1477.6	16,825.8a	4985.3b	2649.0a
	RN40% + HOM	1581.8	1518.8	17,454.1a	5161.2a	2060.6d

^a^Treatment: CK (zero N), FN (farmers’ traditional inorganic N rate),RN (recommended inorganic-N rate), HAN (zinc and humic acid urea), RN40% + HOM (40% inorganic N rate of RN with homemade organic matters). Different letters in the same column within the same crop and rotation system represent significant differences of mean values at the *P* < 0.05 level.

The treatment of RN and HAN in wheat and maize recorded the same management costs as FN, but the output value significantly increased by 2.1% and 12.8% in relation to FN. The output value of RN40% + HOM significantly increased by 14.4% and by 3.5% compared with RN and HAN treatment. Meanwhile, the net income of RN and HAN were significantly increased by 8.5% and 27.1% compared to FN during the period of wheat/maize growth. The use of organomineral and biostimulating fertilizers (RN40% + HOM) increased by 84.0% the fertilizer cost and by 2.8% the management cost as compared to FN in the whole rotation system. It also increased the output value by 16.7%, but the net income was approximately the same as in the FN treatment (Table 5).

## Discussion

Significant correlation between soil N_2_O flux with soil moisture (Figs. 2, 3 and Table 2) indicated that humidity was the mainly limiting factor for elevated N_2_O emissions under N fertilizer increase in the rotation season^32^. The larger amount of fertilizer in the maize field as compared to wheat field, led to rapid increase of nitrification and denitrification processes shortly after fertilization, which in turn stimulated the production of N_2_O, and in combination to increased soil temperature resulted to higher emissions and soil respiration rates, providing positive feedback for microbial metabolism^33,34^. Compared with the peak N_2_O emission after top dressing, the peak N_2_O emission after basal fertilizer application in wheat and maize growing was higher (Figs. 2, 3). It can be related to low N uptake rates by crop early at the beginning of the planting season (i.e. smaller root systems, lower plant internal N demands). And higher unused fertilizer N residues in the soil, released into the atmosphere through nitrification, resulting in a larger peak. It suggests that the amount of nitrogen fertilizer should be reduced at the beginning of the planting season^35^.

Average cumulative N_2_O emissions of N treatments were found in the following decreasing order FN > RN > HAN > RN40% + HOM > CK in wheat or maize. Cumulative N_2_O emissions in RN40% + HOM of the wheat/maize rotation system under organic matter regulations were significantly reduced by 41.0% than RN and reduced by 20.9% than HAN treatment. (Table 2). Our finding indicated that using organomineral and biostimulating fertilizers (HOM) were more conducive to N_2_O emission reduction in farmland (Fig. 2, 3, Table 2). In this study, the soil moisture is less than 40% (Figs. 2, 3), which is lower than the suitable moisture environment (70–90%) for the denitrification process^36^. And this is probably due to the relatively low N availability and application rate of easily decomposable organic C in the HOM treatment did not result in a more favorable environment for denitrification compared with other treatments^36^.

Previous study reported that the use of trichodermaviride biofertilizer combined with chemical fertilizers cut down the discharge amount of N_2_O by 33.3–71.8%^20^, and this is similar to what observed in this study. Moreover, the reduced N_2_O emission under the application of HOM may be due to bacterial and microbes. The growth of microorganisms in organic treatment needs to consume part of the excess nitrogen source, and will release some phenolic substances which affect the activities of nitrifying bacteria in the soil^20^. At the same time, addition of organic matter in this experiment can release a small amount of organic acid, which is beneficial to reduce the soil pH in the test area (test area soil pH = 8.3) and create a more suitable soil environment for nitrifying bacteria (pH = 6.5–7.5)^37^. The specific mechanism needs further study. In support to our findings the comparison among N fertilizer treatments of the N_2_O emission factors (EF) showed that these parameters increased with application amount of N fertilizer (Table 2). EF of the different N treatments in the rotation system in this study varied 0.15–0.38%. These values within the range in the default value of 1% suggested and in line with reported findings^27,34^. The application of RN40% + HOM showed the smallest N_2_O emission coefficient and emission intensity in the whole rotation season, and the emission reduction potential was the largest.

Fertilizer N recovery can be considered as a N balance between crop uptake and microbial N fixation in different soils. The NUE of a crop is the result of interactions between climate, soil conditions, microbes and organic or inorganic nitrogen sources^38^. Therefore, under certain circumstances reduced N fertilizer could increase the NUE of crops and reduce the apparent N loss^8^. Under the conditions of our experiment, the N uptake of crops was slightly increased in the treatments with reduction inorganic N, But N uptake of the grain significantly increased by 28.4–35.7% (Table 3). This suggested that excessive inorganic N fertilizer caused excessive N translocation to the vegetative organs (stems and leaves), reducing grain N uptake and utilization^39^.

N input from sources in FN in the two seasons was greater than the current crop N uptake (132.5 vs. 125.9 kg ha^−1^ for wheat and 257.9 vs. 243.7 kg ha^−1^ for maize, respectively) (Table 4), indicating that the chemical N fertilizer application was not beneficial to soil N balance and raising N losses in the soil–water-atmosphere through N leaching and denitrification^15^. The use of zinc and humic acid urea (HAN) instead of inorganic N fertilizer (RN) under the same N content could significantly increased the NPE and NAE of the crop by 10.0% and 22.4% in rotation system (Table 3). Soil residual N in HAN was closed to RN and the apparent N loss decreased by 14.8% in HAN as compared to RN in maize season (Table 4). This result indicated that HAN was more effective in preventing excessive N loss, which might be related to the humic acid and zinc elements in the treatment. Liu et al^40^ showed using same amount of inorganic nutrients, humic acid fertilizer could improve urease activity of soil, plant N uptake, and additionally increasing the rhizome yield by 19.7%. In agreement with the previous studies, the use of HAN in our experiments resulted in 12.9% increase of grain yield and 564.9 USD ha^−1^ of the net income in the rotation system as compared to FN (Table 5). Compared with RN, the NPE, NAE, and NUE of RN40% + HOM in the rotation system were significantly increased (Table 3). RN40% + HOM fertilizer decreased soil residual N by 22.0% and apparent N loss decreased by 30.1% compared to RN after a rotation (Table 4), whereas, its yield was the highest (Table 5). These results can be attributed to the use of HOM since an organic–inorganic complex through cation exchange and chemical fertilizer nutrient increased the soil nutrient storage capacity and held more N resources, beneficial to soil nutrient balance^41^. Moreover, the organic acid produced in the decomposition process of organic fertilizer also reduces the amount and activity of urease in the surrounding soil. Reducing the activity of microorganisms involved in nitrification and denitrification, thereby reducing the loss of nitrogen and improved NUE^42^. Microbial agent might play a role in improving soil structure, fertility and stable or high yield^43^. Besides, Bai et al^44^ found that the N fertilizer with bacteria could reduce the N input and reduce the N accumulation by 22–29%, which was consistent with our findings.

## Conclusions

In summary, we observed the effects of different nitrogen reduction and combined application of organic material treatments on farmland N_2_O emissions and nitrogen utilization and loss, comprehensively its output and farmers' benefits. The result showed that soil N_2_O emission was significantly correlated with soil moisture and N fertilizer. RN40% + HOM treatment featured the lowest N_2_O emission, it had a positive effect on reducing greenhouse gas emission. During the whole growth period of wheat and maize, the peak N_2_O emissions of base fertilizers relative to topdressing of all treatments were higher. However, the RN40% + HOM treatment can significantly reduce the peak N_2_O emission compared with other treatments. Cumulative N_2_O emissions during the maize growing season were significantly higher than these during the wheat growing season. Compared with RN and HAN treatments, the cumulative emissions of RN40% + HOM treatment was reduced by 41.0% and 20.9%, respectively. Meanwhile, it has the lowest N application rate but the N uptake and yield of grain was the highest, soil residue N and the apparent N loss decreased. But its economic net income was closed to FN. It is thus concluded that application N fertilizer reduced and consisting of homemade oganic matter treatment could be a recommend practice for the North China plain as a win–win solution-high yields and low N_2_O emission. In addition, both RN and HAN treatments are beneficial to decrease N_2_O emissions and increase nitrogen utilization, the net income of RN and HAN were significantly increased by 8.5% and 27.1% compared to FN during the period of wheat/maize growth. These practices can be taken to agricultural regions where organic fertilizers are not readily available.
